# 3D Printed Models for Teaching Orbital Anatomy, Anomalies and Fractures

**DOI:** 10.18502/jovr.v16i4.9751

**Published:** 2021-10-25

**Authors:** Roya Vatankhah, Ali Emadzadeh, Sirous Nekooei, Bahar Tafaghodi Yousefi, Majid Khadem Rezaiyan, Hossein Karimi Moonaghi, Mohammad Etezad Razavi

**Affiliations:** ^1^Department of Medical Education, School of Medicine, Mashhad University of Medical Sciences, Mashhad, Iran; ^2^Department of Radiology, Mashhad University of Medical Sciences, Mashhad, Iran; ^3^Oculoplastic & Strabismus, Khatam Eye Hospital, Mashhad University of Medical Sciences, Mashhad, Iran; ^4^Department of Community and Public Health, Mashhad University of Medical Sciences, Mashhad, Iran; ^5^Nursing and Midwifery Care Research Center, Department of Medical Surgical Nursing, School of Nursing and Midwifery, and Department of Medical Education, School of Medicine, Mashhad University of Medical Sciences, Mashhad, Iran; ^6^Eye Research Center, Mashhad University of Medical Sciences, Mashhad, Iran

**Keywords:** 3D Printed Models, Learning, Ophthalmology Residents, Orbit

## Abstract

**Purpose:**

The aim of this study was to determine the efficacy of using 3D printing models in the learning process of orbital anatomy and pathology by ophthalmology residents.

**Methods:**

A quasi-experimental study was performed with 24 residents of ophthalmology at Mashhad University of Medical Sciences. Each stratum was randomized into two groups. The educational booklets were distributed, and various forms of orbital 3D models were printed from orbital computed tomography (CT) scans. Knowledge enhancement on the topics was measured by comparing pretest and posttest scores.

**Results:**

Thirteen residents who were trained using traditional methods were deemed the control group; while 11 residents who were trained using the 3D printed models were classed as the intervention group. The control group was younger than the intervention group (*P* = 0.047). The results showed that there was a statistically significant difference in the total posttest scores between the two groups. Based on the repeated measures of the analysis of variance (ANOVA), score variables were significant between the two groups (*P* = 0.008). Interestingly, the use of the 3D educational model was more effective and statistically significant with the year one residents as compared to the year two residents (*P* = 0.002).

**Conclusion:**

This study is the first one in Iran quantifying the effects of learning using 3D printed models in medical education. In fact, 3D modeling training is seemingly effective in teaching ophthalmic residents. As residents have never encountered such technology before, their experience using 3D models proved to be satisfactory and had a surprising positive effect on the learning process through visual training.

##  INTRODUCTION 

In the era of communication, innovative changes in the science and technology industry have facilitated greater access to valuable information. As a consequence, the educational system should focus on student-centered structural learning as it pertains to technology, to ensure continued synergy. A place for the teaching-learning process has begun under the idea of technology. The rapid advancement of science is influenced by computer technologies that are used in the education process and will remove the limitations of traditional education.^[1]^

The advent of 3D printing has created new ways to complement the health practice. These exciting new technologies allow for the uniqueness and customized visualization in the production of various imaging process of medical tools and also assist in devising complex and customized objects.^[2]^ When used for specific medical purposes, 3D printing technology is recognized as one of the newest devices in expanding the use of health-related innovations.^[2]^ Loke et al examined the effect of the use of a three-dimensional model on the learning process. The results showed that as a result of the use of the 3D models in training, the pediatric cardiopulmonary residents were motivated to acquire more information about congenital heart disease.^[3]^ The purpose of our study was to determine the impact of learning on ophthalmology residents using 3D printing of models from orbital CT for training purposes.

The effectiveness of applying 3D models for teaching and learning in various subjects indicates the useful application of these models. 3D modeling is effective in improving students' visual-spatial skills in the teaching–learning process.^[4]^ One distinguishing feature of 3D models is that it provides immediate feedback to student, interacting and providing enhanced realistic learning experiences in a clinical setting.^[5]^ Orbit surgery can be challenging due to the compact anatomy of the orbit.^[6]^ Interestingly, 3D models may also be developed for tissue repair in orbital surgery to assist in the prevention of tissue hernias inside the sinuses; in addition, actual 3D models may be used in repairing orbit fractures to cover bone defects.^[7]^ According to the studies, a 3D printing model can be used in ophthalmology as a tool in assisting the restoration of facial bones, especially orbital wall fractures.^[8]^

Although it mentioned that 3D models can help to facilitate the understanding of anatomy and anomalies in conjunction with the use of the 2D images.^[9]^

Understanding particular body parts that have certain complexities such as orbital and other complex mid face bones from using 2D images is indeed complicated. In fact, it is not entirely possible to ascertain with 2D or even 3D images. However, by providing models that are fully compliant with the CT scan, normal-sized dimensions of patients' affected regions would assist in visualizing a better environment from different angles and enabling touch from multiple anatomical areas, thus making it easier for residents to understand the inherent pathologies and anatomies.

Instructional teaching materials should be made available in a manner that is uncomplicated and affordable due to the importance of empowering students to learn concepts effectively through visual aids and touch training, which provides better understanding, as sensory aids may improve memory and the learning process.^[10]^

##  METHODS

This quasi-experimental study evaluated the effect of using 3D models on educational outcomes. The residents were categorized based on their educational stage (namely, first or second year enrollment), and then stratified randomization was performed. However, randomization is more effective when the sample size is higher than 100 in each group, which was not the case in the present study. This issue was considered in the interpretation of results, where it was highlighted in the main text.

The study was performed on the selected ophthalmology residents of Mashhad University of Medical Sciences in two phases. Firstly, a standardized educational booklet was prepared based on the reference source of the ophthalmology (American Academy of Ophthalmology 2017–2018) and secondly, an educational model was designed based on 3D printing obtained from radiological images, including normal orbital CT scans, various types of fractures, and some congenital abnormalities. How to produce 3D models has been discussed in detail in another study.^[11]^

After the randomization was performed based on the residents' academic ID, the subjects from each academic year were divided into two groups defined as the intervention and control group, respectively. The inclusion criteria consisted of the selected ophthalmology residents in the first and second academic years (2019 and 2020). Those who were unwilling to participate in the research were excluded from the study.

The standard training booklet about knowing orbital anatomy and pathology was prepared with the cooperation of specialists in medical education and ophthalmology. The training booklet was given to both the intervention and the control groups to revise the topics related to anatomy, anomalies, and orbital fractures. The level of information retained was measured using various methods of training. The 3D model technique was taught to the intervention group during a 2-hr session. An ophthalmologist provided detailed explanations, and answered questions.

All residents, in both the control and the intervention groups were tested twice at different intervals, firstly, three days after the initial training, residents were tested on their short-term ability to retain information (memorization) and secondly at fourteen days after training to determine their retention of information. The pretest and posttest included 12 multiple choice questions with one-point for correct choices and zero points for wrong choices or unanswered ones.

Pretest and posttest questions were different because we assumed that using identical questions would reduce the responsiveness, and increase recall bias. To ensure that the two tests were analyzed with the same parameters, the discrimination and difficulty index of the test were calculated and reported. The data analyzer was unaware of which of the groups were assigned to the analysis.

Data were described using SPSS software version 16 where a per-protocol design was considered. The quantitative data were displayed in terms of average and standard deviation, while the qualitative data were described in terms of frequency and percentage. Pretest scores followed normal distribution in both the control and the intervention groups, but posttest scores did not follow a normal distribution. The comparison between quantitative variables recorded in the two groups was performed using student *t*-test for the pretest and Mann–Whitney for the posttest at three- and fourteen-day intervals. Qualitative variables in the two groups were evaluated using Chi-square. The trend of the score variables within the two groups was calculated using the repeated measures of ANOVA. The relationship between the use of the studying booklet and the posttest scores was evaluated using the Pearson's correlation test. All tests were two-tailed, and the significance level was less than 0.05.

##  RESULTS 

Among the 24 first and second year residents of ophthalmology, 12 were men (50%); the overall average age was 29.66. Table 1 shows the frequency and number of groups studied by grade, age, and gender. The control group was three years younger than the intervention group (*P* = 0.047) [Table 1].

**Table 1:** Basic characteristics of the studied groups


		**Control group (** ***n*** ** = 13)**	**Intervention group (** ***n*** ** = 11)**	**Significance level**
Gender	Male	8 (61.5)	4 (27.3)	0.093 ${}^{1}$
	Female	5 (38.4)	7 (63.6)	
Age	28 ± 1.5	31.2 ± 3.9	0.047 ${}^{2}$
Academic year	First Year	6 (46.1)	6 (54.5)	0.088 ${}^{1}$
	Second Year	7 (53.8)	5 (45.4)	
${}^{1}$ Probability value test Chi2 ${}^{2}$ Mann–Whitney test Data reported as Mean ± Standard Deviation or Frequency (%)			

**Table 2:** The total scores of the groups studied in the pretest and posttest


	**Control group**	**Intervention group**	**Significance level**
Pretest scores	7.46 ± 1.89	7.90 ± 2.02	0.058*
Posttest scores	Three days	2.25 ± 7.69	10.63 ± 2.15	0.003**
	Fourteen days	8.15 ± 2.19	10.27 ± 2.10	> 0.001**
*T-test **Mann–Whitney test			

**Table 3:** Subgroup analysis based on the academic year


	**Studied groups**	**Academic year**	
	**Year one**	**year two**
Pretest scores	Control group	7.33 ± 1.86	7.57 ± 2.07
	Intervention group	9.16 ± 1.16	6.40 ± 1.81
	*P*-value	0.068*	0.334*
Posttest three days	Control group	7.83 ± 2.13	7.57 ± 2.50
	Intervention group	12 ± 0.0	9.00 ± 2.34
	*P*-value	0.002**	0.285**
Posttest fourteen days	Control group	8.66 ± 1.96	7.71 ± 2.42
	Intervention group	10.66 ± 1.50	9.80 ± 2.77
	*P*-value	0.049**	0.071**
*T-test **Mann–Whitney Data reported as Mean ± Standard Deviation or Frequency (%)			

Thirteen residents were trained traditionally using medical definitions and 1D images for anatomy, fractures, and orbital diseases, while eleven residents were trained using the printed 3D model technique. The difficulty index of the pretest was 0.76, its discrimination index was 0.34, the difficulty index of the posttest was 0.76, and its discrimination index was 0.60. The results showed a statistical difference between the total posttest scores in the control and intervention groups [Table 2].

**Figure 1:**
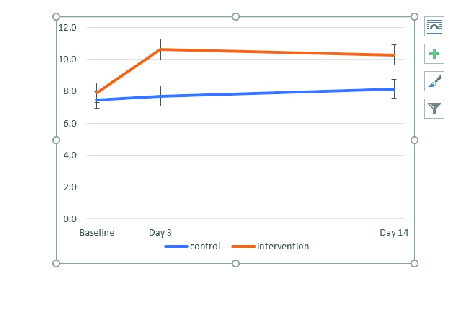
Changes in scores in the two study groups along with error bars (1 SE)

Figure 1 illustrates the variables in scores in the two study groups along with error bars (1 se).

The trend of score variables based on the repeated measures of ANOVA was significant between the two groups (*P* = 0.008).

The study of the linear relationship between the score variables at the baseline, then at three days and fourteen days posttest was performed using the Pearson's correlation test. The changes in scores from baseline to three days was positively correlated with the number of pages studied (*r* = 0.416, *p* = 0.043). In addition, the change in score at three days and fourteen days positively correlated with the number of pages studied (*r* = 0.487, *p* = 0.016). The amount of the pages of the educational booklet studied three times did not differ significantly between the two groups.

In the subgroup analysis based on the residents' academic year in the whole study, the 3D training intervention had significantly better results in one-year residents. Interestingly, in this study the 3D educational model was found to be more effective and statistically significant in junior (year one) residents rather than their senior counterparts [Table 3].

##  DISCUSSION

The purpose of this study was to determine the impact on learning of ophthalmology residents using 3D printing models from orbital CT scans. It was observed that the intervention of 3D training through models was influential in motivating residents to learn. Based on the total scores, the findings of the present study showed that the educational model was more applicable and effective in the intervention group, which was statistically more significant than the control group. Figure 1 shows the changes in the scores, which can be generalized throughout the community. This figure suggests perpetual learning from the control group, but the intervention group, which possessed the same baseline as the control baseline, increased their scores and then decreased significantly during the time interval between three days after the posttest and fourteen posttest were justified.

The use of 3D models for training process has many positive effects on learning. Loke et al (2017) found that the learning intervention group of cardiac assistants as compared to the control group were effectively taught the 3D model technique of Fallot tetralogy disease.^[3]^ Montgomery (2019) asserted that 3D modeling of complex fractures in orthopedic assistants' training programs could be a valuable training tool, especially for first-year assistants.^[12]^ In the same regard, the Weinmann 2014 study also found that 3D technology had a meaningful impact on facilitating and developing influential learning environments, which was effective in creating positive learning experiences and motivation in students. In addition, 3D printers were essential tools in the development of people's visual spatial intelligence.^[13]^ Canessa referred to 3D printers as essential tools in increasing interest in the classroom, also in developing efficient learning activities for teaching conceptual information or complex situations.^[14]^ Lütolf also stated that students' motivation in contributing to the development phase of the 3D project was on the rise, as they are fully interested in the project.^[15]^ Blikstein proposed that their 3D printers were effective in teaching and increasing motivation, performance, and knowledge retention in students.^[16]^ Slavkovsky also found 3D printers to be successful in teaching social studies courses.^[17]^ Also, Krassenstein noted that the production of abstract or rigid educational materials, especially in the social sciences (e.g., geography, history, geology), has important implications for further understanding of compelling learning experiences.^[18]^

The findings of the present study showed that there was a statistically significant difference between the two groups after 14 days of follow-up. The difference between the mean score of the control and intervention groups indicated that the intervention group scores were higher than the control scores. Thus, learning with physical models inherently influences and potentially enhances the knowledge of assistants, consistent with the 2015 study by Yammine et al.^[19]^ The Sisson 2012 study also showed a 3D interactive learning technique to facilitate learning and long-term knowledge with ancillary teaching materials.^[20]^ The Lim et al 2018 study revealed three-dimensional models were considered by orthopedic assistants to be a desirable training method which indicated increase in accurate diagnoses of tabular fractures.^[21]^ In summary, the use of 3D teaching materials can positively affect in the learning of 3D understanding of orbital and midfacial anatomy and anomalies.

These educational models were considered to be able to sense for better understanding and visualization. The varied benefits of 3D touch training models include abstract reinforcement of points and content, stimulation of the imagination, enhancement of perception, increased student retention, expanded memory capacity, and increased attention and focus in the classroom.^[22]^ According to the literature reviewed, this is the first study in Iran to determine the effect on learning through the use of 3D printed models in medical education as it relates to specialized ophthalmology residency. Residents who experienced multiple levels of training were all given access to the same materials and assessments. It was determined that 3D printing makes it possible to convert medical images, computed tomography (CT), into tangible models thereby providing solutions to address particular issues such as dealing with orbital bone, fissures, and the complex anatomy of the orbit. As residents had never encountered such technology before this study and have had no such experience before, it has been proven that the investigation of the use of 3D models in training of complex anatomical and pathological body structures was successful.

This method of teaching may have surprising positive effect on the trainee's visual and perceptive competencies as well as the whole learning process.

##  Financial Support and Sponsorship

The authors thank the Research and Educational Chancellor of Mashhad University of Medical Sciences for the material-spiritual support, in particular EDC.

##  Conflicts of Interest

None declared.

##  Appendix

The project implementation of the 3D printing-based model is shown as follows;^[11]^ (A written consent was taken from the residents to publish theirs photo.)








